# Advancements in 3D Transoesophageal Echocardiography (TOE) and Computed Tomography (CT) for Stroke Prevention in Left Atrial Appendage Occlusion Interventions

**DOI:** 10.3390/jcm13226899

**Published:** 2024-11-16

**Authors:** Reza Hajhosseiny, Ben Ariff, Graham Cole, Michael Koa-Wing, Punam Pabari, Nilesh Sutaria, Norman Qureshi, Prapa Kanagaratnam, Bushra Rana

**Affiliations:** 1 National Heart and Lung Institute, Imperial College London, London, W12 0HS, UK; graham.cole3@nhs.net (G.C.); prapa.kanagaratnam@nhs.net (P.K.); 2 Imperial College Healthcare NHS Trust, Hammersmith Hospital, London, W12 0HS, UK; b.ariff@nhs.net (B.A.); michael.koa-wing@nhs.net (M.K.-W.); punam.pabari@nhs.net (P.P.); nsutaria@nhs.net (N.S.); norman.qureshi@nhs.net (N.Q.); bushra.rana@nhs.net (B.R.)

**Keywords:** LAAO, left atrial appendage, stroke, anticoagulation, CT, TOE, 3D

## Abstract

Left atrial appendage occlusion (LAAO) has emerged as a highly effective alternative to oral anticoagulation for stroke prevention in patients with non-valvular atrial fibrillation. Precise pre-procedural planning and meticulous post-procedural follow-up are essential for achieving successful LAAO outcomes. This review explores the latest advancements in three-dimensional (3D) transoesophageal echocardiography (TOE) and computed tomography (CT) imaging modalities, which have considerably improved the planning, intra-procedural guidance, and follow-up processes for LAAO interventions. Innovations in 3D TOE and CT imaging have transformed the approach to LAAO by providing a more detailed and accurate assessment of the left atrial appendage, enabling clinicians to acquire comprehensive anatomical and morphological information, crucial for optimising device selection and positioning, thus reducing the risk of complications and enhancing the overall safety and efficacy of the procedure. Post-procedurally, CT and TOE imaging are invaluable in the monitoring of patients, ensuring that the device is correctly positioned and functioning as intended. Early detection of any complications (e.g., device-related thrombus and peri-device leaks) can help to risk-stratify patient at increased risk of stroke and initiate timely interventions, thereby improving long-term outcomes for patients.

## 1. Introduction

Atrial fibrillation (AF) is the most frequently observed cardiac arrhythmia in clinical practice, with a high risk of morbidity and mortality, imposing a significant burden on healthcare systems [1]. The risk rises significantly with age, from 1.5% in people aged 50–59 to 23.5% in those aged 80–89 [2], and it is estimated that globally, over 59 million individuals were living with AF in 2019 [3]. This represents a significant increase from 2010, when the prevalence was 33.5 million. However, the actual prevalence of AF is likely higher than these figures suggest, as many individuals remain undiagnosed until patients develop noticeable symptoms.

Patients with AF have an up to 5-fold increased risk of ischaemic stroke, and AF-related ischaemic stroke accounts for up to 24% of all ischemic strokes [4]. The pathophysiology involves the formation of thrombi in the left atrium, particularly within the left atrial appendage (LAA), due to the impaired and disorganised atrial contractility and blood stasis. When these thrombi embolise, they can obstruct cerebral arteries, leading to ischaemic strokes. Strokes associated with AF are often more severe and carry a higher risk of recurrent events compared to strokes of other aetiologies [4,5].

### 1.1. Stroke Prevention in AF

Anticoagulation therapy is a cornerstone in the management of AF to reduce the risk of stroke irrespective of rhythm control strategies [6]. Medications such as warfarin and direct oral anticoagulants (DOACs) significantly decrease the incidence of thromboembolic events by preventing clot formation in the atria [6,7,8,9,10]. However, not all patients can tolerate long-term anticoagulation due to unacceptable risk of bleeding, whilst other patient cohorts may require additional stroke prevention despite adequate compliance with anticoagulation therapies.

For these patients, left atrial appendage occlusion (LAAO) offers an effective alternative or adjunctive treatment. LAAO devices mechanically close off the left atrial appendage, the primary site of thrombus formation in AF, thereby reducing the risk of stroke. Clinical trials have demonstrated that LAAO is non-inferior or comparable to warfarin and DOACs in preventing stroke [2,11,12,13], particularly in those at higher risk of bleeding. Integrating LAAO into treatment protocols for patients who are intolerant to anticoagulation or require additional protection can potentially optimise stroke prevention strategies and improve overall patient outcomes.

### 1.2. Types of LAAO

**Figure 1:**
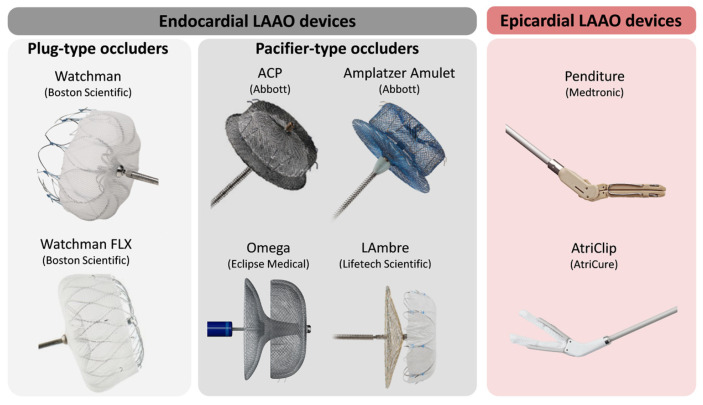
Different designs of percutaneous left atrial appendage devices. The Watchman and Watchman FLX devices extracted from Boston Scientific Corporation (USA) or its affiliates. The Amplatzer Cardiac Plug (ACP) and Amplatzer Amulet, from Abbot Corporation (USA). LAmbre device from Lifetech Scientific Corporation (China). Omega device from Eclipse Medical Corporation (Ireland). Penditure device from Medtronic Corporation (USA). AtriClip device from AtriCure Corporation (USA). LAAO: left atrial appendage occlusion; ACP: Amplatzer Cardiac Plug. Adapted with permission from Albors et al., 2024 [14].

Left atrial appendage occlusion (LAAO) devices are designed to prevent thrombus formation in the left atrial appendage (LAA), thereby reducing the risk of stroke in patients with atrial fibrillation (AF). The primary types of LAAO devices include the endocardial systems (Watchman, Amplatzer Amulet and Omega) and the epicardial systems (Figure 1) [14].

The Watchman device is one of the most widely utilised percutaneously implanted LAAO devices. It is a parachute-shaped, self-expanding structure made of nitinol mesh, plugging the LAA to prevent clot formation. Clinical trials such as PROTECT-AF and PREVAIL have demonstrated its efficacy and safety [2,11].

The Amplatzer Amulet device is another percutaneously implanted choice, featuring a dual-seal mechanism with a lobe and a disc that occludes the LAA opening, providing a secure and complete closure. The AMULET IDE trial has highlighted its effectiveness and safety compared with the Watchman, whilst complete LAA occlusion was significantly higher for the Amulet occluder than for the Watchman device [13]. 

The Omega occluder is a self-expanding device implanted percutaneously, featuring a cup-and-disc configuration crafted from a seamless nitinol wire mesh coated in platinum to seal off the LAA [15]. A flexible waist connects the cup and disc, with the disc containing polypropylene fabric stitched inside to strengthen its occlusive properties. The cup includes 6 to 10 hooks for secure anchoring and device stability.

The epicardial systems (e.g., LARIAT system) involve a suture or clip delivery system that ligates the base of the LAA externally, thereby excluding it from the circulation. This method is particularly advantageous for patients who may not be candidates for implantable devices.

### 1.3. Left Atrial Appendage Anatomy and Morphology

The LAA is an anatomically distinct, finger-like extension from the left atrium, which is located on the anterosuperior surface of the left atrium [16]. It has three distinct regions, (1) the ostium, which is the opening to the left atrium; (2) the neck; and (3) the body, which has a varying number of lobes. The LAA varies considerably in shape and size among individuals [17]. Its walls are composed of pectinate muscles, which create a trabeculated interior surface [17]. The unique structure of the LAA, with its trabeculated muscle and variable lobes, predisposes it to blood stasis and thrombus formation, making the LAA a primary source of emboli that can lead to stroke. 

**Figure 2:**
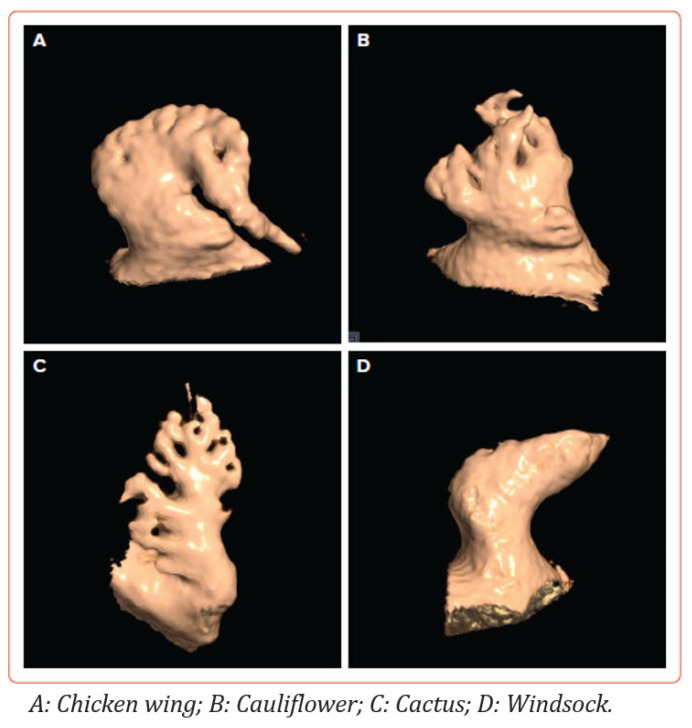
Four different left atrium appendage morphologies as shown by CT. Adapted with permission from Dudziñska-Szczerba et al. [18].

The LAA is typically classified into several morphological types: “chicken wing”, “cactus”, “windsock”, and “cauliflower”, best seen on computed tomography (CT) (Figure 2) [16,17,18]. The chicken wing morphology, which is the most common type (up to half of patients) characterised by a prominent bend or fold in the appendage, is generally associated with a lower risk of stroke [19]. In contrast, the cactus morphology (up to 30% of patients) with its multiple lobes, and the cauliflower morphology (least common type), with a more complex, shorter, and broader appearance, are considered higher risk for thrombus formation [20]. The windsock morphology (up to 20% of patients), with its single dominant lobe and variable length, presents an intermediate risk [20].

Understanding these morphological and anatomical variabilities of the LAA, including differences in its length, volume, and the number of lobes, has significant implications for both surgical and percutaneous interventions aimed at occluding the LAA. Advanced imaging modalities, such as transoesophageal echocardiography (TOE) and CT, are essential for accurately assessing LAA anatomy and morphology to guide anticoagulation therapy and selecting appropriate LAAO devices.

## 2. Pre-Implantation Imaging

### 2.1. Cardiac CT

Cardiac CT has emerged as an important imaging modality for the assessment and suitability for patients for LAAO. With its ability to provide exceptionally high isotropic spatial resolution, high temporal resolution, and a wide field of view, CT allows for comprehensive 3D multiplanar reconstruction for pre-implantation planning. The added benefit of being non-invasive makes it a safe choice for patients, while its accessibility ensures it can be readily deployed across a wide range of clinical settings.

#### 2.1.1. Anatomical Contraindications

Cardiac CT is particularly useful for assessing the anatomical contraindications to LAAO. The most common absolute contraindication is LAA thrombus. This is seen as a hypoattenuating filling defect within the appendage, which persists on delayed phase imaging, distinguishing it from slow flow [16]. In a meta-analysis of cardiac CT versus TOE for the detection of LAA thrombus, the combination of first pass imaging with delayed phase imaging resulted in a significantly improved diagnostic accuracy with a sensitivity and specificity of 100% and 99%, respectively, and a positive predictive value and negative predictive value of 92% and 100%, respectively [21].

**Figure 3:**
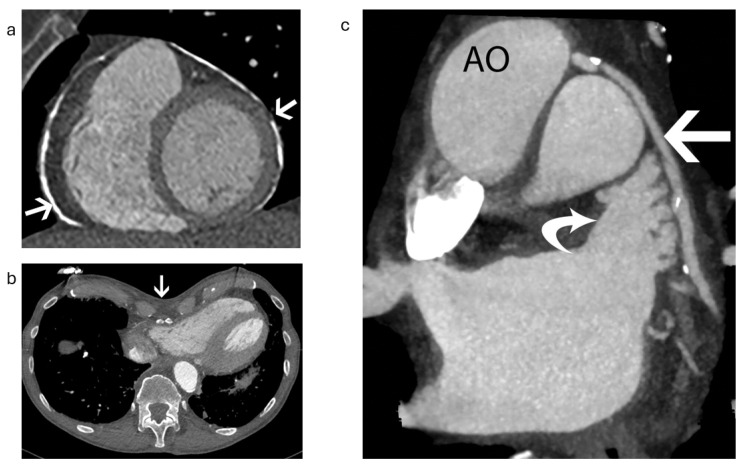
Contraindications for placement of hybrid endocardial–epicardial devices in three patients. AO stands for aorta (**a**) Short-axis reconstructed CT image in a 47-year-old man shows circumferential pericardial calcification (arrows). (**b**) Axial CT image in a 43-year-old man shows a severe pectus excavatum deformity (arrow) with a Haller index score of 4.5. (**c**) Coronal reconstructed CT image in a 72-year-old man shows a venous bypass graft from the aorta to the obtuse marginal artery (straight arrow), which is situated directly adjacent to the LAA (curved arrow). CT: Computed tomography, LAA: Left atrial appendage. Adapted with permission from Rajjah et al. [16].

Additional contraindications, particularly for the insertion of hybrid endocardial–epicardial devices (such as the LARIAT system), include conditions such as pericarditis, pericardial adhesions, pectus excavatum, and a posteriorly rotated heart, with the LAA situated either behind the pulmonary trunk or next to a coronary bypass graft [16]. All of these conditions can be clearly identified through cardiac CT imaging prior to implantation (Figure 3) [16].

#### 2.1.2. LAA Measurements

**Figure 4:**
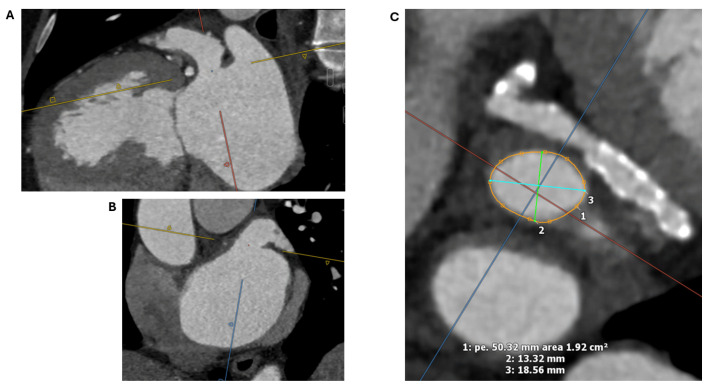
Cardiac CT measurement of the LAA ostium. Using 3D multiplanar reconstruction, cutting a double oblique plane across the coumadin ridge and the left circumflex artery (**A**,**B**) creates an en-face view of the ostium (**C**), determining its shape, perimeter (1), minimum (2) and maximum (3) diameters, as well as area (1). CT: Computed tomography; LAA: Left atrial appendage; 3D: Three dimensional.

The predominant measurements for LAA device implantation are the LAA ostial dimensions, device landing zone dimensions, and LAA length. The LAA is defined postero-superiorly by the well-distinguished coumadin ridge, which separates the LAA from the left superior pulmonary vein. The LAA is defined antero-inferiorly by the thin wall of the left atrium, which separates the LAA from the mitral valve annulus. The ostium should be measured at its largest in the systolic phase of the cardiac cycle (30–40% of R-R interval). Using 3D multiplanar reconstruction, cutting a double oblique plane across the coumadin ridge and the left circumflex artery creates an en-face view of the LAA ostium, determining its shape, perimeter, maximum and minimum diameters, as well as area (Figure 4).

**Figure 5:**
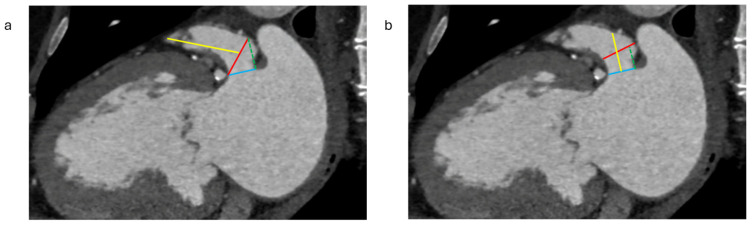
(**a**) Measuring the landing zone for the Watchman device. The landing zone is positioned 10–20 mm within the LAA (dotted green line), starting from the Coumadin ridge. A line that connects the LAA near the left circumflex artery to the point 10–20 mm inward from the Coumadin ridge is then drawn (red line) to create an en-face view of the landing zone. The measurement of the dominant lobe length is taken from the plane of the landing zone to the tip of the dominant lobe (yellow line). LAA: Left atrial appendage. (**b**) Measuring the landing zone for the Amplatzer amulet device. The landing zone is positioned 10–12 mm inside the LAA orifice (dotted green line). The diameters are then determined using an en-face-view image, captured at this location, with measurements taken perpendicular to the LAA walls (red line). The LAA depth is assessed from the orifice (blue line) to the roof of the LAA (yellow line). LAA: Left atrial appendage.

The optimum landing zone is device-specific and should be measured in accordance with manufacturer instructions and guidelines [22]. However, general principles can be followed for each device [16]. For the Watchman device, the landing zone is typically positioned 10–20 mm within the LAA, starting from the Coumadin ridge. To measure the landing zone diameters for the Watchman device, an en-face-view image is used, drawing a line that connects the LAA near the left circumflex artery to a point 10–20 mm inward from the Coumadin ridge (Figure 5a). For the Amplatzer Amulet device, the landing zone is positioned 10–12 mm inside the LAA orifice. The diameters are then determined using an en-face-view image, captured at this location, with measurements taken perpendicular to the LAA’s walls (Figure 5b).

The length of the dominant lobe plays a crucial role in determining the appropriate device size [16]. A minimum length is necessary to prevent the implanted device from protruding into the LA cavity. Inadequate LAA length also increases the risk of device embolization and pericardial effusion. Manufacturer guidelines provide specific minimum LAA length requirements for each device. For the Watchman device, the measurement of the dominant lobe length is taken from the plane of the landing zone to the tip of the dominant lobe (Figure 5a). It is important that this length exceeds the maximum diameter of the landing zone. For the Amplatzer Amulet devices, the LAA depth is assessed from the orifice to the roof of the LAA (Figure 5b). A depth of more than 10–12 mm is recommended to ensure the safe placement of these devices.

Whilst these measurements can often be highly accurate and reproducible, there is currently a wide degree of variation in implementation across cardiac centres depending on local expertise and protocols. Whilst most centres combine the measurements derived from cardiac CT in conjunction with those obtained intra-procedurally from TOE to select the most appropriate device size, some other centres with expertise in intra-cardiac echocardiography (ICE) rely on the CT measurements, avoiding the need for TOE and a general anaesthetic.

#### 2.1.3. Assessment of the Inter-Atrial Septum

Cardiac CT enables comprehensive assessment of the inter-atrial septum and detection of pathologies which can potentially impact LAA closure. These conditions encompass defects in the atrial septum, a patent foramen ovale, aneurysms in the septum, thickening due to lipomatous hypertrophy, and the presence of cardiac tumours.

**Figure 6:**
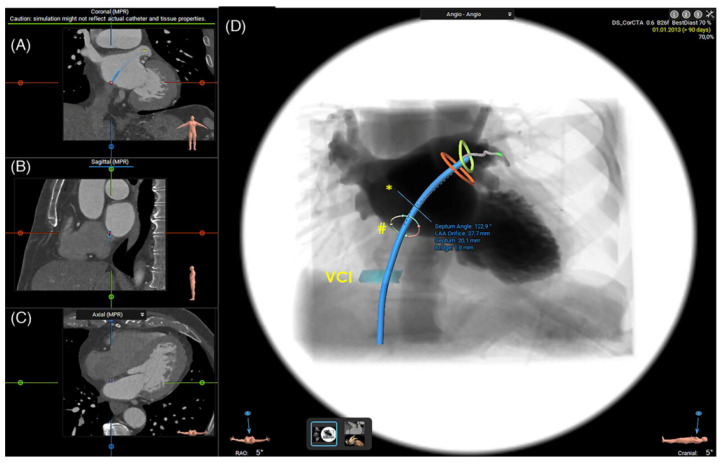
Measurement of the angle between the transseptal puncture site and LAA ostium. (**A**–**C**) Simulated transseptal crossing in the coronal (**A**), sagittal (**B**), and axial (**C**) plane. (**D**) Virtual Angio view with a visualization of the angle between IAS and LAA. Green circle—LAA landing zone; Orange circle—LAA ostium; Hash sign—Projected septum contour with a simulated inferior/posterior puncture; Asterisk—Projected septum length and angle. LAA: Left atrial appendage. Adapted with permission from Nelles et al. [24].

Furthermore, to reduce the risk of device tilting or peri-device leakage (PDL) and to limit the need for catheter exchanges, it is crucial to select a puncture site that enables a straight trajectory along the LAA’s long axis, perpendicular to the ostium. Cardiac CT and dedicated post-processing software can be used to guide the interventionalist by simulating the optimal septal puncture site, guidewire, and device catheter trajectory to enable interactive rehearsals of the LAAO procedure, enhancing procedural familiarity (Figure 6) [23,24].

#### 2.1.4. Other Anatomical Considerations

The shape and morphology of the LAA has a direct impact on procedural success and complexity. Whilst the “windsock”- and “cactus”-type LAAs enable the most straightforward measurement and device selection, the “chicken wing”-type appendage is more challenging and increases procedural complexity due to the short length of the LAA near the bend and the challenges of manoeuvring the catheter around this acute bend [16]. Cardiac CT can help guide the interventionalist with prior knowledge of the LAA anatomy and morphology, enabling optimal device selection and procedural strategy.

The left superior pulmonary vein is used to anchor the guidewire and deliver the catheter before the LAA closure device is introduced into the LAA. Therefore, the anatomical relationship between the LAA and the left superior pulmonary vein is crucial for procedural success [16].

Prior knowledge of the left atrial dimensions can help guide the interventionalist with regard to the catheter’s distance from the atrial septum to the left superior pulmonary vein and LAA. 

Finally, important anatomical structures such as the left circumflex artery, great cardiac vein, venous bypass grafts, persistent left superior vena cava, sinoatrial nodal artery originating from the left circumflex artery, the left phrenic nerve, the left internal mammary artery, and the inferior epigastric artery can all be found in close proximity to the LAA and can be impacted during the procedure. Cardiac CT can provide a rapid 3D assessment of these adjacent structures to the LAA.

#### 2.1.5. 3D Printing and Virtual Reality

**Figure 7:**
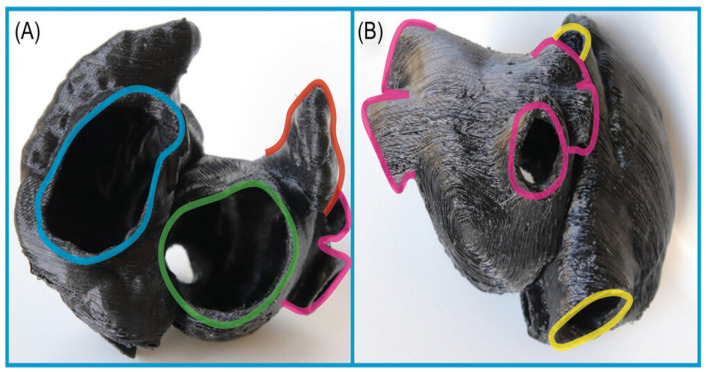
Three-dimensionally printed model. (**A**) Anterior view, (**B**) posterior view; blue, tricuspid annulus; green, mitral annulus; pink, pulmonary veins; red, left atrial appendage; yellow, superior vena cava and inferior vena cava. Adapted with permission from Hozman et al. [25].

**Figure 8:**
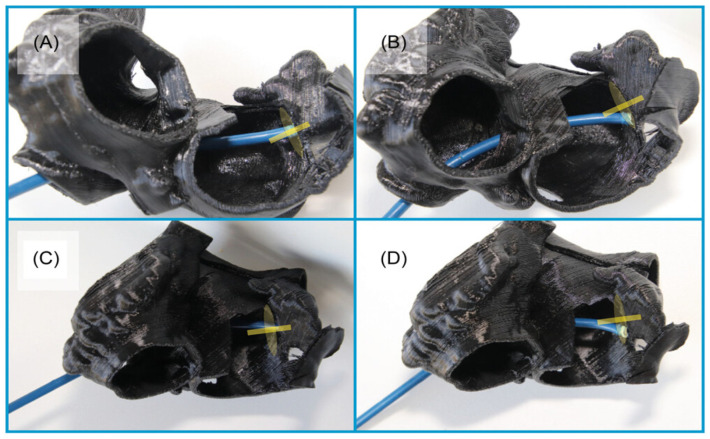
In vitro TSP simulation. LAA ostium and its axis are marked in yellow. (**A**,**B**) left anterior oblique (LAO) projection, (**C**,**D**) LAO with cranial angulation; (**A**,**C**) optimal sheath position (posterior TSP), (**B**,**D**) nonoptimal sheath position (anterior TSP). LAO, left anterior oblique; TSP, transseptal puncture. Adapted with permission from Hozman et al. [25].

The integration of 3D printing and virtual reality (VR) technologies with CT imaging data have been developed for pre-procedural planning for LAAO procedures [25]. Patient-specific 3D-printed models of the LAA and surrounding structures can be created, allowing clinicians to physically examine the anatomy and simulate the procedure (Figure 7 and Figure 8) [25].

### 2.2. Transoesophageal Echocardiography

Transoesophageal echocardiography (TOE) provides real-time visualization of the LAA before, during, and after device implantation, offering a valuable alternative for patients who may not be suitable for CT imaging. Unlike CT, TOE does not require ionizing radiation or contrast agents, making it safer for patients with renal impairment, contrast allergies, or other contraindications to contrast-based imaging [26].

#### 2.2.1. LAA Thrombus Assessment

**Figure 9:**
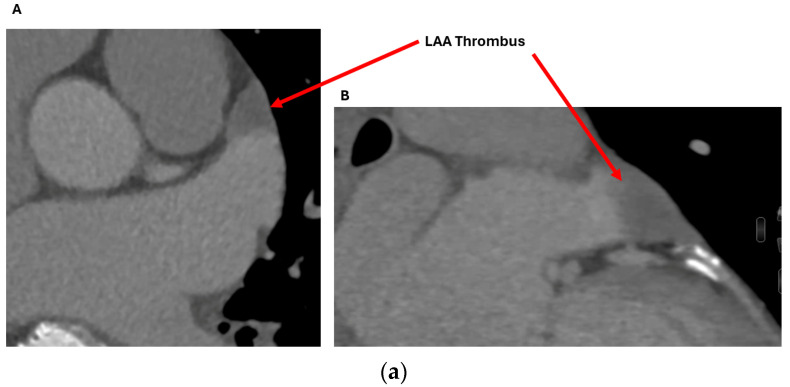

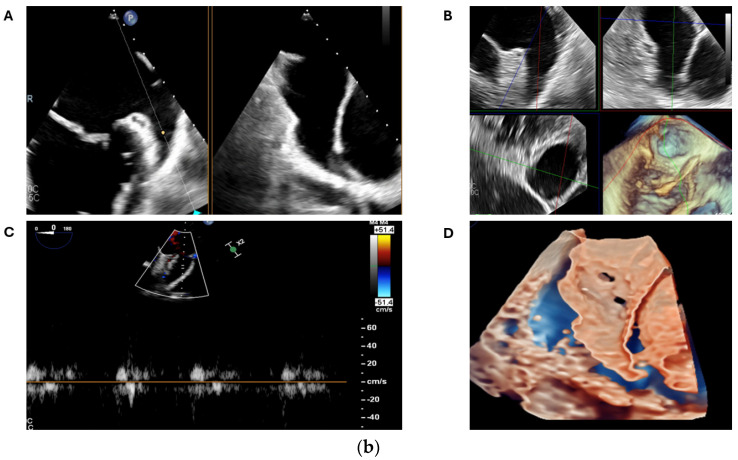
(**a**) A 70-year-old gentleman with atrial fibrillation on an appropriate dosage of warfarin was found to have a LAA thrombus on cardiac CT. Despite increasing the target INR range to 3-4, the filling defect persisted on repeat cardiac CTs (delayed phase shown here in images (**A**,**B**)), with a Hounsfield Unit of consistently < 50 HU. (**b**) The patient underwent a TOE to further assess the LAA for the presence of a persistent thrombus despite optimal anticoagulation. (**A**) X-plane 2D TOE showing a possible spontaneous echo contrast in the distal LAA, also seen on the 3D multiplanar reformat (**B**). (**C**) Pulsed-wave velocity assessment of the LAA showed a doppler velocity of 17 cm/s, in keeping with slow flow or spontaneous echo contrast. However, on the 3D glass view analysis (**D**), the entire appendage is clearly visualised with no evidence of thrombus, highlighting the importance of advanced 3D TOE as the gold standard imaging modality for the assessment of LAA thrombus. The patient underwent successful LAAO with no complications, and follow-up imaging showed no evidence of PDL or DRT. TOE: Transoesophageal echocardiogram; LAA: Left atrial appendage; LAAO: Left atrial appendage occlusion; 2D: Two-dimensional; 3D: Three-dimensional.

Whilst cardiac CT is highly accurate at excluding a LAA thrombus, TOE remains the gold standard and final arbiter in complex cases [27,28]. With the ability to deliver real-time 2D and 3D anatomical depictions of the LAA, in combination with colour and pulsed-wave doppler assessment of the blood flow patterns within the LAA and the use of advanced image processing within modern echocardiography machines, operators can confidently observe the presence or absence of LAA thrombus (Figure 9a,b) [27,28].

#### 2.2.2. LAA Measurements (Anatomy and Morphology)

**Figure 10:**
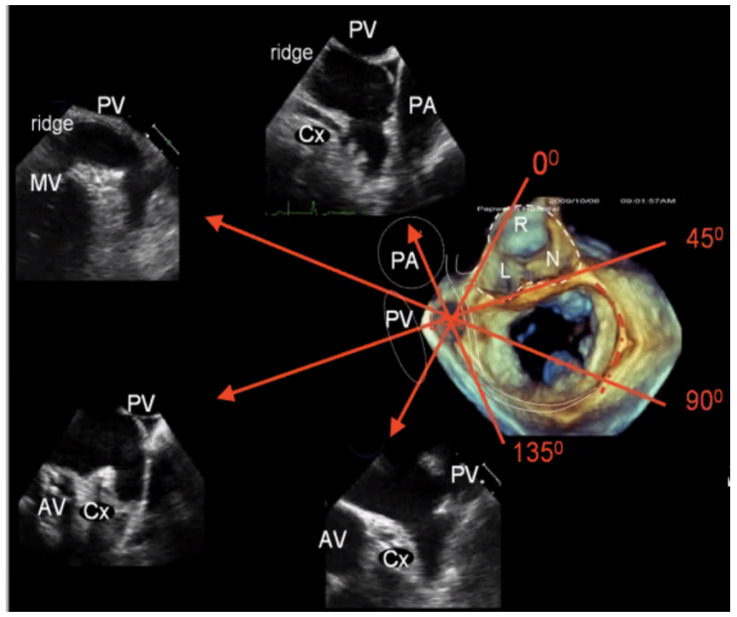
Conventional 2D transoesophageal echo view (0, 45, 90, and 135°) of the left atrial appendage and surrounding structures. AV: Aortic valve; MV: Mitral valve; PA: Pulmonary artery; PV: Pulmonary vein; Cx: Left circumflex artery.

**Figure 11:**
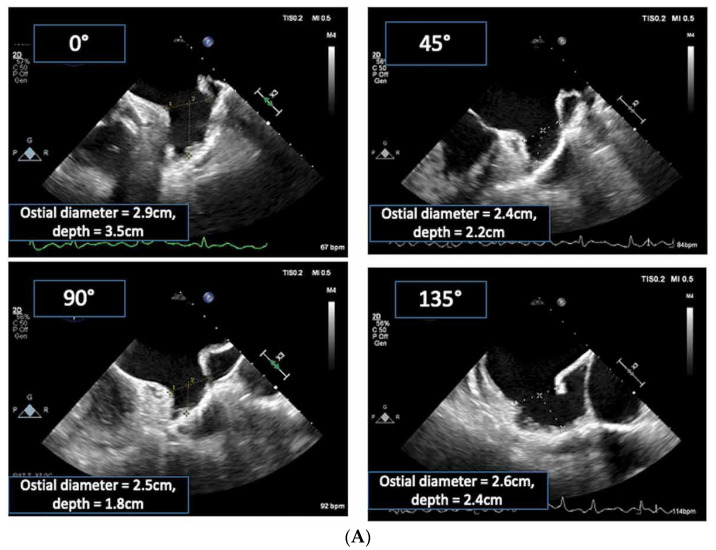

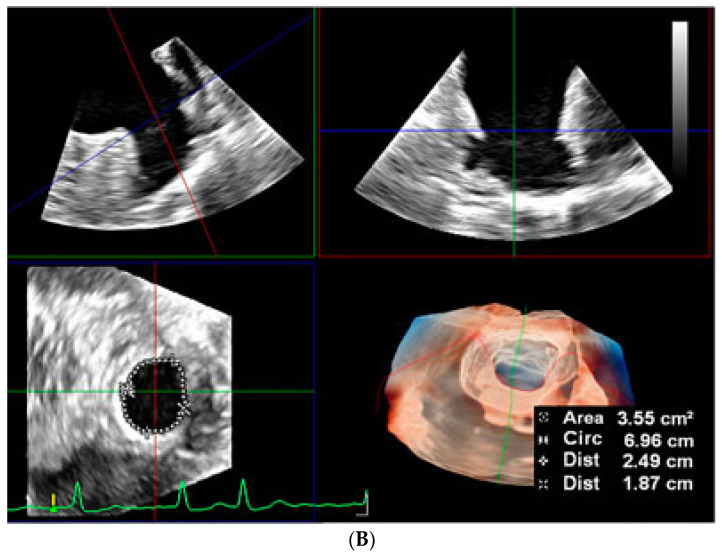
(**A**) Transoesophageal echo (TOE) image showing the potential device landing zone in the 0°, 45°, 90°, and 135° views. It should be measured from the origin of the circumflex artery to the roof of the LAA at least 10–20 mm below the ligament of Marshall/coumadin ridge. Depth is measured from the plane of landing zone to the LAA apex. Adapted with permission from Ramchand et al. [29]. (**B**) Three-dimensional LAA measurement. MultiVue mode is employed on the LAA 3D Zoom image, and at the end of the systole, the landing zone positioning line (blue line) in the LAA sagittal plane (**upper left**) and coronal plane (**upper right**) are adjusted to obtain the LAA landing zone cross-section (**lower left**), and the LAA LZ maximum diameter (the first Dist value obtained), minimum diameter (the second Dist value), area, and circumference (Circ) are manually measured. The **lower right** figure shows 3D downward view of the LAA LZ and the measurement results. Adapted with permission from Sun et al. [27].

As with cardiac CT, TOE is able to directly assess the LAA’s ostial dimensions, the device landing zone’s dimensions, and the LAA’s length [26]. With 2D TOE, the recommended orthogonal views are 0°, 90°, 45°, and 135° to fully appreciate the shape and dimensions of the LAA (Figure 10). The LAA ostium is measured from the origin of the left circumflex artery to the tip of the coumadin ridge in multiple views. The landing zone for the device is assessed from the left circumflex artery to the roof of the LAA, leaving a minimum 10 mm gap (Figure 11A) [22,29]. However, despite obtaining these orthogonal views, 2D TOE may not fully appreciate the complex 3D shape and orientation of the LAA. Indeed, in a single-centre study of cardiac CT versus 2D TOE, using 2D TOE alone would have resulted in 62% of patients being undersized for their device [30]. 

**Figure 12:**
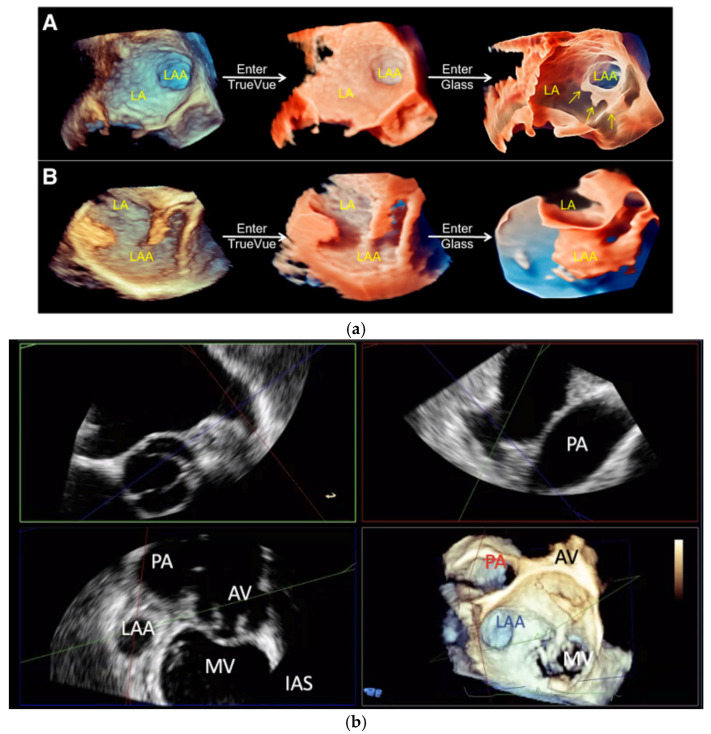
(**a**) Conversion of conventional real-time 3D transoesophageal echocardiography to TrueVue Glass. (**A**) From left to right, conventional 3D, TrueVue, and TrueVue Glass imaging modes of the LAA at the entrance of the left atrium are shown. The TrueVue Glass (right) shows the three lobular structures at the blind end of the LAA (arrows). (**B**) From left to right, conventional 3D, TrueVue, and TrueVue Glass imaging of the LAA in lateral view are shown. 3D: three-dimensional; LA: Left atrium; LAA: Left atrial appendage. Adapted with permission from Sun et al. [27]. (**b**) Three-dimensional (3D) multiplanar reformat images depicting the anatomical relationship of the LAA with the surrounding structures as seen with 3D TOE. AV: Aortic valve; MV: Mitral valve; PA: Pulmonary artery; LAA: Left atrial appendage; IAS: Inter-atrial septum; TOE: Transoesophageal echocardiogram.

**Figure 13:**
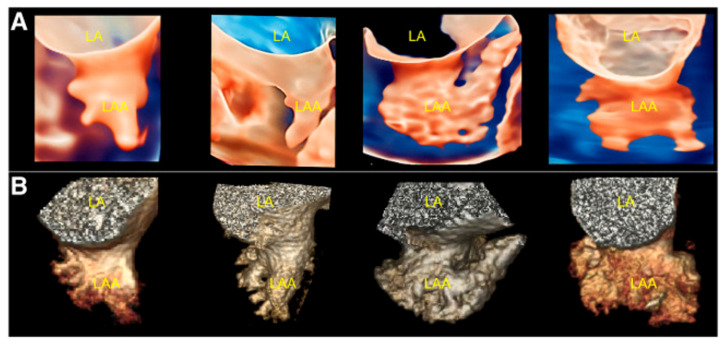
Comparison of TrueVue Glass and CT for displaying different morphologies of LAA. (**A**) From left to right, TrueVue Glass shows cactus, windsock, chicken wing, and cauliflower types of LAA. (**B**) From left to right, CT display of the above morphological types of LAA are shown. CT: Cardiac computed tomography; LA: Left atrium; LAA: Left atrial appendage. Adapted with permission from Sun et al. [27].

Whilst echocardiography intrinsically under-sizes the LAA compared to cardiac CT, largely due to the fasting hydration status of the patient, the introduction of 3D TOE has significantly improved the precision and depth of LAA assessment (Figure 11B) [22,27]. Unlike traditional 2D TOE, which can be limited by its planar view and angle dependencies, 3D TOE provides a fuller perspective on the anatomy, morphology, and spatial relationships of the LAA with adjacent cardiac structures (Figure 12a,b and Figure 13) [27]. This enhanced view allows clinicians to better appreciate complex anatomical details, such as the shape, volume, and orientation of the LAA, which are critical for accurate diagnosis, risk stratification, and planning for LAAO. This added level of detail from 3D imaging may offer a closer approximation to what is observed with cardiac CT, bridging the gap between the two modalities and supporting more informed clinical decision-making.

**Figure 14:**
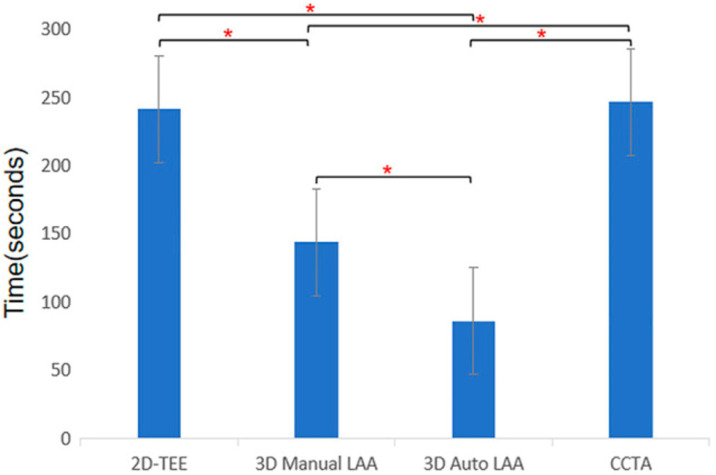
Time consumption of different methods for measuring morphological parameters of left atrial appendage. 2D-TEE, two-dimensional transoesophageal echocardiography; 3D Auto LAA, three-dimensional automated left atrial appendage; 3D Manual LAA, three-dimensional manual left atrial appendage; CCTA, cardiac computed tomographic angiography; * *p*-value < 0.05. Adapted with permission from Sun et al. [27].

Furthermore, 3D multi-planar reformat reconstruction enables full circumferential assessment of the LAA orifice and landing zone, thereby measuring the minimum and maximum diameters. Indeed, in a direct head-to-head comparison of 2D TOE with 3D TOE and with cardiac CT as a reference, 3D TOE was significantly more accurate than 2D TOE and comparable to CT [27,31,32]. Real-time 3D TOE with artificial intelligence capability has been shown to increase the accuracy, reproducibility, and efficiency of LAA assessment compared with 2D TOE and cardiac CT (Figure 14) [27]. However, to our knowledge, no direct head-to-head comparative study has evaluated the long-term outcomes of 3D TOE versus cardiac CT-guided LAAO in terms of device complications and stroke.

#### 2.2.3. Intra-Procedural TOE

An advantage of TOE over cardiac CT is that it can assess the suitability of LAA closure and guide the interventionalist within the same session. Similar to cardiac CT, 2D and 3D TOE can assess the inter-atrial septum for septal defects, a patent foramen ovale, septal aneurysms, lipomatous hypertrophy, and cardiac tumours. A trans-septal puncture is then performed under hybrid TOE and fluoroscopic guidance, with the guide wire directed to and anchored in the left upper pulmonary vein [26]. The optimal puncture site can be selected under 2D TOE, with the superior/inferior localisation seen best in the bicaval view (90–110°), and the anterior/posterior localisation seen best in the mid-oesophageal aortic valve short axis view (25–45°) [26]. Simultaneous multi-planar views can obtain both views at the same. In experienced centres, 3D TOE assessment of the septum and trans-septal puncture has shown promising results [33]. 

Once the closure device is guided into its final position and deployed under TOE and fluoroscopy, 2D and 3D TOE with real-time colour doppler can be used to assess the device position, stability and the presence of peri-device leaks (PDLs) before and after release [34]. The presence of a residual iatrogenic atrial septal defect can also be documented. These images can subsequently be used for accurate comparison at follow-up.

### 2.3. Intracardiac Echocardiography (ICE)

Intracardiac echocardiography (ICE) has gained traction as a viable alternative to TOE in intraprocedural imaging for LAAO, given its unique advantages in providing real-time, high-resolution imaging from within the cardiac chambers [35]. Studies have shown that ICE imaging yields comparable accuracy to TOE in guiding trans-septal puncture, device positioning, and device deployment in LAAO [35,36]. Unlike TOE, which typically requires general anaesthesia or deep conscious sedation, ICE can often be performed with or without sedation. This reduction in anaesthesia need is particularly advantageous in patients with comorbidities where anaesthesia risk is high. It is also a valuable alternative in situations when TOE is contraindicated, e.g., gastroesophageal varices/tears or bleeds.

Moreover, 3D and 4D ICE, which offer real-time volumetric imaging, represent the latest advancements, combining the advantages of ICE with enhanced spatial resolution and live, dynamic visualisation of cardiac structures [37,38,39]. Preliminary studies suggest that 4D ICE may further improve procedural guidance by providing real-time, multi-planar reconstructions of the LAA and other key anatomic landmarks without the need for operator-controlled rotations as required in 2D ICE [39]. Although still emerging, 4D ICE shows promise in improving the accuracy of LAAO procedures by enabling real-time, high-resolution imaging from within the cardiac chambers. 

However, ICE has limitations, including the need for specialised catheters and expertise, as well as potential challenges in visualising the posterior structures due to its intracardiac perspective. Furthermore, ICE can be associated with higher per patient equipment costs compared to TOE, although this is likely offset by reducing the need for general anaesthesia. Finally, in a recent large comparison study, the use of ICE was associated with significantly higher rates of pericardial effusion compared with TOE [40].

## 3. Post-Implantation Imaging

Once the closure device has been deployed, follow-up imaging is required to assess its position, function, and effectiveness. It is recommended to wait at least 45 days post-deployment before assessment, which is the typical time required for device endothelialisation [16,27]. The main reported complications following LAAO are stroke, device embolization, peri-device leak (PDL), device-related thrombus (DRT), pericardial effusion, and device erosion. Both CT and TOE are used for post-procedural follow-up imaging, either individually or in conjunction.

### 3.1. Device Position and Embolization

**Figure 15:**
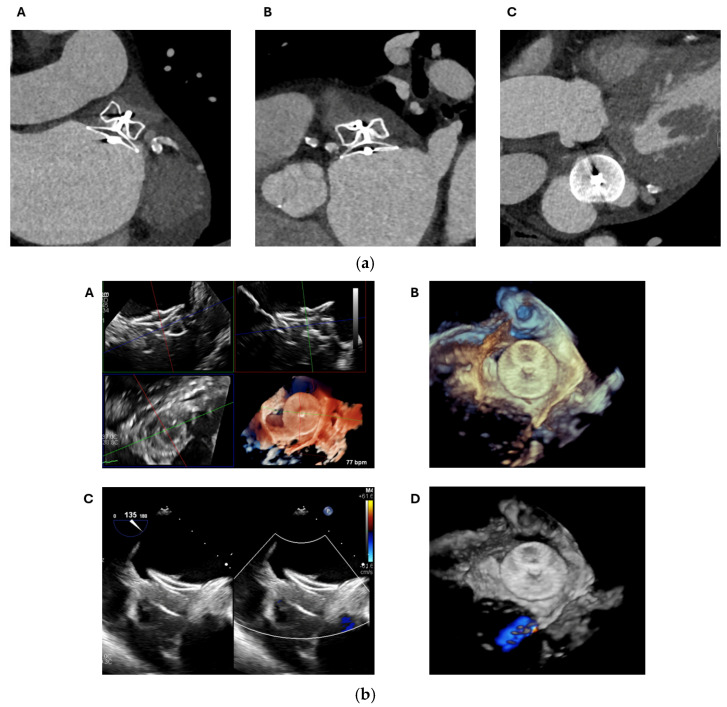
(**a**) A 71-year-old gentleman imaged with cardiac CT 8 weeks post LAAO with an Amplatzer Amulet device. The 3D multiplanar reformat shows a well-positioned device with no evidence of embolization, as seen on the two orthogonal views (**A**,**B**) as well as the short-axis view (**C**), PDL or DRT. (**b**) The same patient was also imaged with TOE. This confirmed a well-positioned device on 3D multiplanar reformat (**A**) and 3D zoom (**B**), with no evidence of embolization or DRT. 2D colour doppler with 135° view shown here (**C**) and 3D zoom with colour doppler (**D**) showed no evidence of PDL. LAAO: Left atrial appendage occlusion, TOE: Transoesophageal echocardiogram, 2D: Two-dimensional, 3D: Three-dimensional, CT: Computed tomography, PDL: Peri-device leak, DRT: Device related thrombus.

CT and TOE are essential imaging modalities for assessing the position and potential embolization of LAA closure devices. They both provide high-resolution 3D images that allow for precise visualisation of the device within the LAA, enabling evaluation of its orientation, deployment, and ensuring the device is securely positioned and effectively sealing the LAA, reducing the risk of stroke and thromboembolism (Figure 15).

Device embolization is a relatively rare but serious complication, with reported rates of 0.26% for the Watchman device and 0.2% for the newer-generation Amplatzer Amulet device [41,42]. Risk factors include inappropriate device sizing, non-coaxial alignment, and inadequate depth of placement. LAA-related risk factors include complex anatomical variations, a wide ostium, an enlarged landing zone, and insufficient LAA depth [16]. The vast majority of device embolization occurs during or immediately after implantation, and therefore TOE plays an important role in the diagnosis and percutaneous/surgical device retrieval. However, delayed and often asymptomatic device embolization has been reported up to 12 months after implantation, particularly for smaller device sizes and CT can play an important role in the diagnosis and localisation of these late presenting device embolizations [41,43].

### 3.2. Peri-Device Leaks

Peri-device leaks (PDLs) are relatively common post LAA closure; they are described in up to 57.3% of patients [44]. Risk factors include a complex LAA anatomy (windsock-shaped LAA; oval/elliptical-shaped ostium and landing zone); large LA size; failure of endothelialisation; off-axis positioning; and failure of full device expansion on deployment [16,45]. Conventionally, a PDL is categorized based on its size; minimal (<1 mm); mild (1–3 mm); moderate (4–5 mm); or severe (>5 mm). When the leak exceeds 5 mm in size; it is considered significant; as it may compromise the effectiveness of the device and increase the risk of thromboembolic stroke [16,44,45,46]. Detection and classification of such leaks is crucial for determining the appropriate course of action, which may include further intervention (e.g., plugging the gap) or continued oral anticoagulation therapy.

However, classification based on PDL size alone might be too simplistic, and it has recently been shown that even minimal PDL sizes can be significantly associated with a thromboembolic event [44]. Instead, risk stratification should be based on a combination of the type of imaging modality used to detect the PDL, its absolute size, and the mechanism of leak in relation to the type of device deployed [44,46]. For example, a PDL tract around a plug occluder (e.g., Watchman device) might represent a different degree of thromboembolic risk compared to a disc-and-lobe occluder (e.g., Amplatzer Amulet device), where the PDL may indicate blood flow between the left atrium and the appendage beyond the lobe, or flow between the left atrium and the area between the disc and lobe [46,47].

**Figure 16:**
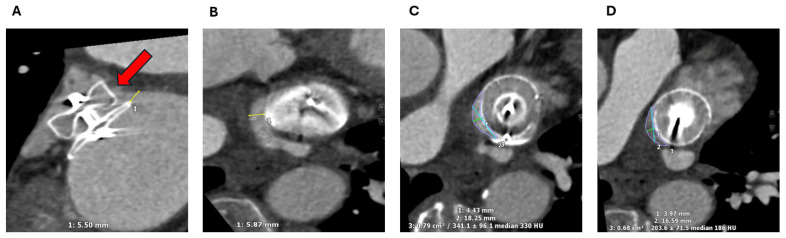
A 73-year-old woman post LAAO with an Amplatzer Amulet device. Multiplanar cardiac CT images show a clear antero-superior PDL tract between the LA and the distal LAA (red arrow) (**A**). The tract measures up to 6 mm at the level of the disc (**A**,**B**). At the mid-lobe level (**C**), the tract measures 4 × 18 mm with a cross-sectional of area of 0.79 cm^2^. At the distal edge of the lobe (**D**) it measures 4 × 17 mm with a cross-sectional of area of 0.68 cm^2^. The Hounsfield Units in the distal LAA measures 276 HU, compared with the LA of 336 HU (i.e., more than 25% and 100 HU). LA: Left atrium; LAA: Left atrial appendage; LAAO: Left atrial appendage occlusion; CT: Computed tomography; PDL: Peri-device leak; HU: Hounsfield Units.

With cardiac CT, a PDL is defined as a continuous visible tract of contrast opacification around the device and into the LAA (Figure 16). Complete occlusion on CT is defined as an absence of a visible contrast tract around the device with a radiodensity of <100 Hounsfield Units (HU) in the LAA and/or <25% of the contrast opacification of the left atrium [48]. Therefore, continued opacification of the LAA distal to the device even in the absence of a directly visualised tract meets the definition of a PDL on CT. The clinical significant of these types of PDLs on CT is uncertain but could represent a fabric leak due to failure of endothelialisation or so called “micro-leaks” [46]. For this reason, the prevalence of PDL is significantly higher on CT compared to TOE with less certain prognostic significance [44]. 

**Figure 17:**
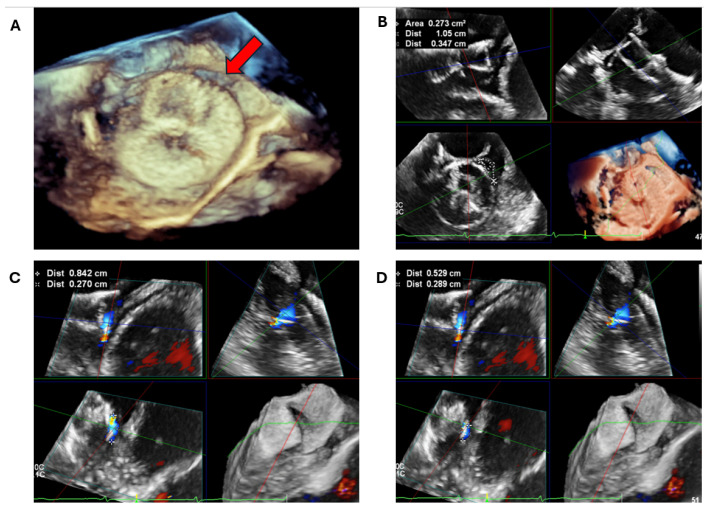
The same patient in Figure 16 was assessed with TOE. The antero-superior PDL tract can be visualised on the 3D zoom image (red arrow) (**A**). This measures 3 × 11 mm with a cross-sectional area of 0.3 cm^2^ at the level of the disc on 3D multiplanar analysis (**B**). On 3D colour doppler analysis, high-velocity flow can be visualised into the distal LAA, which measures 3 × 8 mm at the level of the mid-lobe (**C**) and 3 × 5 mm at the level of the distal lobe (**D**). TOE: Transoesophageal echocardiogram; 3D: Three-dimensional; PDL: Peri-device leak; LAA: Left atrial appendage.

On TOE, a PDL is detected by measuring the width of the colour doppler jet on 2D planes (0, 45, 90, and 135°) or the full dimensions of the colour doppler on 3D imaging [44] (Figure 17). For this reason, the prevalence of PDL on TOE is significantly less than on CT [44]. However, a PDL detected on TOE (regardless of its size) is significantly associated with major adverse events (all-cause mortality and thromboembolism), with a graded positive association with increasing PDL size [44].

### 3.3. Device-Related Thrombus

Device-related thrombus (DRT) is conventionally defined as the presence of thrombus identified on the left atrial aspect of the LAA closure device. It is a relatively uncommon complication of the procedure, with a pooled incidence of 3.8% (351/10,153) in a meta-analysis [49]. Risk factors include a deeper device implantation, permanent AF, higher CHA2DS2-VASc score, large left atrial diameter, and low ejection fraction [16,50,51].

The association between DRT and thromboembolic events is uncertain, with inconsistent reports in the literature. In the PROTECT AF and PREVAIL follow-up, DRT was associated with a significantly increased risk of systemic embolism and ischaemic stroke, with an adjusted rate ratio of 3.55; 95% CI, 2.18–5.79; *p* < 0.001 and 3.22; 95% CI, 1.90–5.45, *p* < 0.001, respectively [51]. Similar findings were observed in pooled registries and meta-analysis reports, with up to 5-fold increased rates of ischemic events in those with DRT [49,52]. However, in the EWOLUTION real-world registry follow-up of patients with the Watchman device, there were no notable differences in the yearly incidence of stroke, TIA, or systemic embolism between patients with DRT and those without (1.7% per year for DRT vs. 2.2% per year for no-DRT, *p* = 0.8) [53]. Similarly, follow-up data from the Amulet IDE trial showed no significant difference in the rates of ischemic stroke or systemic embolism in those with or without DRT [3.1% ± 2.2 vs. 2.6% ± 0.4, hazard ratio 1.15 (95% CI, 0.28–4.73) *p* = 0.85], although overall cardiovascular mortality was significantly increased in those with DRT [8.7 ± 3.4 vs. 3.9 ± 0.5, hazard ratio 2.33 (95% CI, 1.01–5.39) *p* = 0.04] [54].

**Figure 18:**
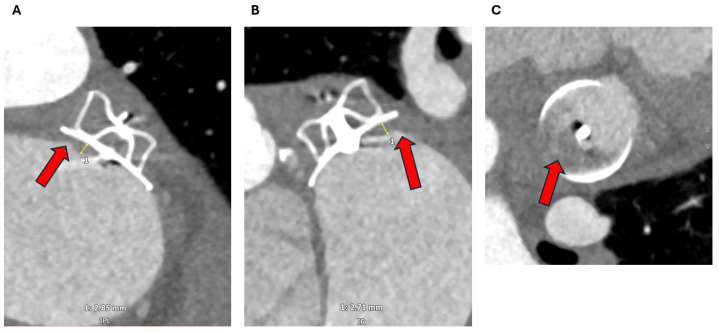
A 72-year-old gentleman imaged with cardiac CT post-LAAO with an Amplatzer Amulet device. A laminar hypoattenuated thickening (HAT) (red arrows) partially covers the antero-superior aspect of the device disc (**A**–**C**) and is continuous with the LA endothelium. It measures 3 mm at its maximum thickness (**A**,**B**). These features are in keeping with a low-grade HAT and likely represent the endothelialisation process. CT: Computed tomography; LA: Left atrium; LAAO: Left atrial appendage occlusion.

Part of the uncertainty stems from a lack of universal imaging definition of DRT, with considerable variability in LAAO surveillance imaging. On cardiac CT, DRT detection has been adapted from the experience of aortic valve replacement imaging, with a hypoattenuated thickening (HAT) on the atrial side of the device. Recently, standardized CT protocols have been introduced to streamline image acquisition and interpretation [50,55]. This involves an electrocardiographically gated cardiac CT with contrast timing optimized for the left atrium (ideally with bolus tracking) and a delayed phase acquisition (between 1 and 3 min post-injection) to enable equilibration of contrast throughout the heart, reducing mixing artifacts near the surface of the device [50,55]. Characteristic features of HAT (HAT/left atrium attenuation ratio, HAT thickness, HAT cross-sectional surface area, laminar vs. pedunculated HAT, and continuation with the left atrial wall) can be used to risk-stratify cases into low-grade or high-grade HAT [55]. Despite this, differentiating DRT from endothelialisation on CT remains very challenging, with histological data supporting the theory that some forms of laminar HAT may be part of the normal healing process (Figure 18) [55,56,57,58].

**Figure 19:**
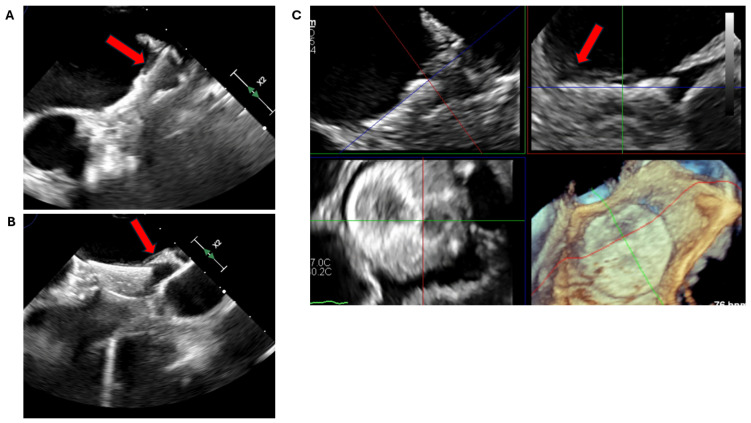
The same patient in Figure 18 was assessed with 2D (**A**,**B**) and 3D TOE (**C**). This shows a well-organised, thin laminar layer of echo-bright lesion partially covering the disc on the atrial aspect of the device (red arrows), in continuity with the LA endothelium and in keeping with normal endothelialisation. TOE: Transoesophageal echocardiogram; 2D: Two-dimensional; 3D: Three-dimensional; LA: Left atrium.

Assessment of DRT on TOE can be equally challenging. In addition to the standard 2D views (0, 45, 90, and 135°), 3D TOE can provide additional informative views and an overview of the shape, depth, and relation of any potential DRT with the device and surrounding structures (Figure 19). An expert group on a retrospective analysis of the PROTECT-AF trial devised five criteria for the identification of DRT on TOE [59]. These included an echo density on the LA aspect of the device (1) not explained by imaging artifact; (2) inconsistency with normal healing/device incorporation; (3) visible in multiple TOE planes; (4) in contact with the device; and (5) exhibiting independent motion. However, this definition is not universal, highly operator dependant, and may not apply to other devices (e.g., Amplatzer Amulet).

Pragmatically, and until a standardized imaging definition and protocol is developed, when DRT is clinically suspected, the current recommendations are to continue/resume oral anticoagulation and monitor the evolution of DRT with serial imaging; often, a combination of CT and TOE can be complementary [50]. In a meta-analysis, re-initiation of anticoagulation resulted in DRT resolution in 97% of patients [49].

### 3.4. Pericardial Effusion and Device Erosion

**Figure 20:**
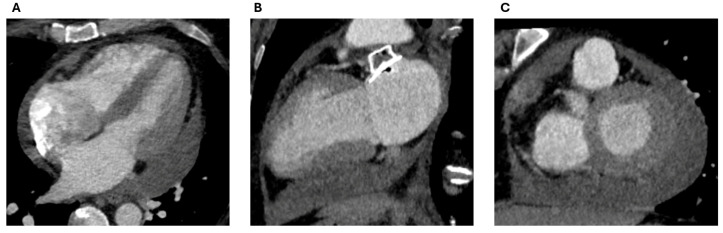
A delayed presentation of pericardial effusion (**A**–**C**) in a 41-year-old woman who was imaged 8 weeks post-LAAO (Amplatzer Amulet device).

Pericardial effusions following LAAO remain uncommon. In a National Cardiovascular Data Registry (NCDR) LAAO Registry, pericardial effusions occurred in 881/63,355 patients (1.35%) [60]. It is usually as a result of direct injury to the thin-walled LAA, its surrounding structures, or the transeptal puncture, and the majority of effusions occur within 24 h of the procedure [61]. However, late presentation effusions have also been described and these are possibly due to chronic injury of the anchoring hooks to the LAA in combination with continued anticoagulation or antiplatelet therapy (Figure 20). Additional risk factors encompass advanced age, female gender, left ventricular function, paroxysmal atrial fibrillation, history of bleeding, lower serum albumin levels, use of dual antiplatelet therapy before the procedure, the number of delivery sheaths used, and the application of double-closure devices [60,62].

Device erosions are extremely rare but can result in serious complications, with reported cases of delayed presentation with tamponade and device migration/protrusion into the adjacent pulmonary artery [63].

Echocardiography remains the gold standard imaging modality for the assessment of pericardial effusions and tamponade; however, incidental pericardial effusions and the precise anatomical location of device erosion in relation to adjacent structures can be identified on CT.

### 3.5. Epicardial Devices

**Figure 21:**
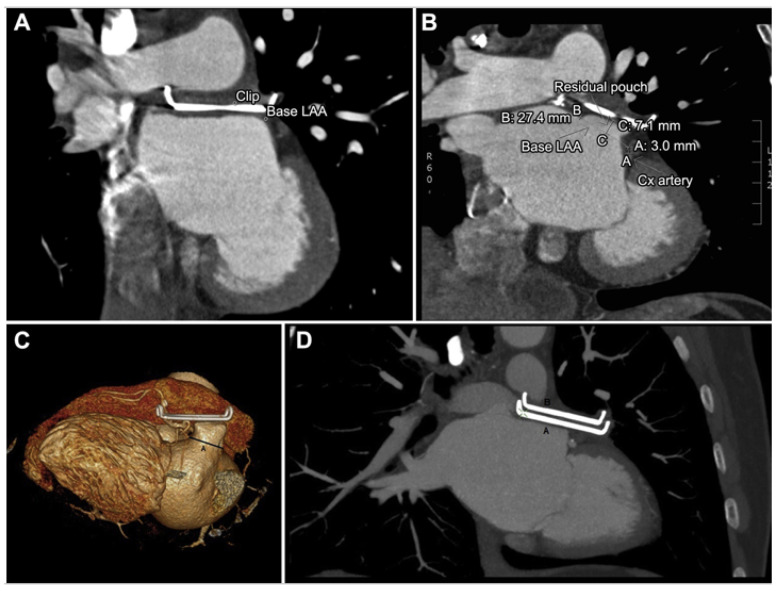
CT assessment of post-surgical LAA epicardial clip closure. (**A**) Example of optimal clip placement. (**B**) Residual left atrial appendage (LAA) pouch measurements after clip placement on the coronal sections. A indicates the base left atrial appendage (imaginary line); B indicates the circumflex (Cx) artery, 3 mm; and C indicates the residual pouch length, 7.1 mm in this illustration. (**C**) Example of a residual pouch of >10 mm. A indicates the position where the clip ideally should have been placed. (**D**) Example of the patient requiring a second clip. A and B are the 2 clips placed. Adapted with permission from Van Laar et al. [64].

CT imaging is highly effective for evaluating the position and closure integrity of epicardial clips, ensuring complete occlusion of the LAA without residual leaks, which could otherwise pose a thrombotic risk (Figure 21) [64]. Additionally, CT allows for precise assessment of the clip’s position relative to the LAA orifice and surrounding structures, facilitating early detection of potential complications such as migration or compression of adjacent anatomy (Figure 21) [64]. This makes CT a particularly useful tool for post-procedural follow-up and long-term management of patients with epicardial devices.

## 4. Conclusions and Future Directions

Percutaneous LAAO is growing as a viable alternative or complementary strategy for stroke prevention in patients with AF, particularly those who are unsuitable for long-term anticoagulation therapy. The pre-procedural imaging approach, which may involve CT or TOE with 3D capabilities, plays a critical role in patient selection and accurate device sizing, ensuring optimal outcomes. During the procedure, imaging guidance using ICE or TOE should be tailored based on the expertise of the clinical team, the availability of resources, and specific patient characteristics.

However, there is a need for a standardized and universally accepted protocol for post-procedural surveillance imaging to monitor device placement, assess for complications such as PDL and DRT, and ensure long-term efficacy. To address this, multicentre randomized controlled trials directly comparing post-procedural CT and TOE are required, providing valuable insights into the most effective imaging modality for follow-up and helping to establish standardized outcome measures that can guide future clinical practice.

## Data Availability

Not applicable.
